# Direct spectroscopic observation of matter falling onto a compact stellar object

**DOI:** 10.1126/sciadv.aef6686

**Published:** 2026-09-18

**Authors:** Roi Rahin, Ralf Ballhausen, Nazma Islam, Maurice A. Leutenegger, Ehud Behar, Daisy Zamora, Joel Coley, Natalie Hell, Tim Kallman, Maximilian Lorenz, Pragati Pradhan, Joern Wilms, Aafia Zainab

**Affiliations:** ^1^ Astrophysics Science Division, NASA Goddard Space Flight Center, Greenbelt, MD 20771, USA.; ^2^ Center for Space Science and Technology, University of Maryland Baltimore County, Baltimore, MD 21250, USA.; ^3^ Department of Astronomy, University of Maryland, College Park, MD 20742, USA.; ^4^ Manipal Centre for Natural Sciences, Manipal Academy of Higher Education, Manipal 576104, India.; ^5^ Department of Physics, Technion - Israel Institute of Technology, Haifa 3200003, Israel.; ^6^ Physics Department, US Naval Academy, Annapolis, MD 21402, USA.; ^7^ Department of Physics and Astronomy, Howard University, Washington, DC 20059, USA.; ^8^ Physics Division, Lawrence Livermore National Laboratory, Livermore, CA 94550, USA.; ^9^ Dr. Karl-Remeis Sternwarte and Erlangen Centre for Astroparticle Physics, Friedrich-Alexander Universität Erlangen-Nürnberg, Bamberg Park 96049, Germany.; ^10^ Department of Physics and Astronomy, Embry-Riddle Aeronautical University, Prescott, AZ 86301, USA.

## Abstract

GX 301–2 is an accreting x-ray pulsar with a massive hypergiant companion WRAY 977, embedded within a dense circumstellar environment. We report on detected redshifted hydrogen- and helium-like iron lines, together with transitions from lower ionization states, observed with XRISM/Resolve during an x-ray flare. Notably, these spectral features evolve from absorption lines at the beginning of the observation to emission lines at the end, with intervening phases exhibiting a mixture of both absorption and emission features. This behavior constitutes direct evidence of inflowing material accreting onto the neutron star. The results suggest a dynamic accretion geometry, evolving from predominantly radial inflow to a transverse, potentially disk-like configuration during the x-ray flare. Observations of inflowing matter thus constitute a direct test of accretion hypotheses and highlight the complex interplay between stellar wind and transient accretion disks in wind-accreting systems.

## INTRODUCTION

Mass accretion is understood to be the powerhouse of x-ray emission in bright x-ray binaries. Among those, high-mass x-ray binaries (HMXBs) are systems consisting of a massive main-sequence or evolved star and a compact object (neutron star or black hole), which accretes directly from the wind of its stellar companion. The basic concept of spherically symmetric accretion of gas has been suggested in the 1940s (*1*–*3*). X-ray emission powered by accretion onto a neutron star was proposed for Sco X-1 in 1967 (*4*), yet many details regarding the mechanism of accretion from stellar winds of massive stars remain unclear despite decades of extensive research (*5*–*8*).

The stellar winds in massive stars ($M_{\text{star}}>8M_{⊙}$) are radiation driven, mostly through ultraviolet lines (*9*, *10*), but their structure is highly inhomogeneous (*11*–*15*). In binary systems, the wind is further perturbed by the orbital motion, gravitational potential, and x-ray emission of the compact object, leading to effects such as bow shocks or local overionization in the wind. In systems with highly magnetized neutron star accretors, the inner accretion flow is further governed by the magnetic field and channeled toward the magnetic poles of the neutron star. Observational diagnostics of the properties of the stellar winds and the accretion flow have thus far mostly relied either on probing large-scale structures through time-resolved high-resolution spectroscopy (*16*–*18*) or the response of the broadband continuum and magnetic features to changes in the mass accretion rate (*19*–*21*), probing the region of immediate deceleration of the infalling plasma.

Observational diagnostics of the accretion flow in between these two extreme scales, the coupling of structures in the stellar wind to the magnetosphere are much scarcer. Specifically, clear indications of wind matter falling onto a compact object have never been observed. Probing the inflowing matter found within the accretion radius requires x-ray observations of absorption lines of highly ionized plasma, with spectral lines of highly ionized iron between 6.5 and 7 keV being the best candidates. Constraining the kinematics of different plasma components allows us to disentangle the accretion flow in the vicinity of the compact object from large-scale structures in the wind, while simultaneous changes in x-ray luminosity probe the accretion timescales of the inflow.

### GX 301–2 XRISM Observation

In this work, we report on our detection of redshifted iron absorption lines in a XRISM observation of GX 301–2. These lines are the first direct spectroscopic observation of accretion onto a neutron star, made possible by the spectral resolution and effective area of XRISM/Resolve (*22*). GX 301–2 hosts a slowly rotating pulsar with a period of P∼685 s embedded in the strong wind of the B1 hypergiant Wray 977 [$∼10^{-5}M_{⊙}{\text{year}}^{-1}$,$v_{\infty }\approx 300$ km s^−1^ (*23*, *24*)]. The neutron star orbit is highly eccentric (ϵ∼0.462), with a period of ∼41.5 days, and the system exhibits regular flares before periastron, between orbital phases 0.85 and 0.95, and near apastron, around phase 0.5 [e.g., (*25*–*29*)]. During the preperiastron flare, both the x-ray luminosity and absorbing column density increase by up to an order of magnitude; the near-apastron flares are more moderate. The accepted model explaining the observed flares is a narrow stream of dense stellar wind, caused by the gravitational influence of the neutron star, which it crosses before periastron and near the apastron (*24*, *29*, *30*). In addition to pulsations from the rotation of the pulsar, the luminosity during flares fluctuates on short timescales not associated with the pulsar’s rotation. These fluctuations are hypothesized to be caused by variations in the accretion rate during the flare, with “off-states” caused by temporary cessation of accretion (*28*). Due to the moderate spectral resolution in the 6- to 7-keV band of x-ray space telescopes such as Chandra/HETG and XMM-Newton/EPIC-pn and EPIC-MOS, previous observations of GX 301–2 focused on the fluorescence lines, especially the prominent neutral iron Kα emission line and its Compton shoulder (*28*, *31*). We note that the GX 301–2 system has a very high column density (*29*), which absorbs almost all photons below ∼2 keV. This makes soft x-ray instruments such as XMM/RGS and Chandra/LETG ill-suited for studying this system.

**Fig. 1.**
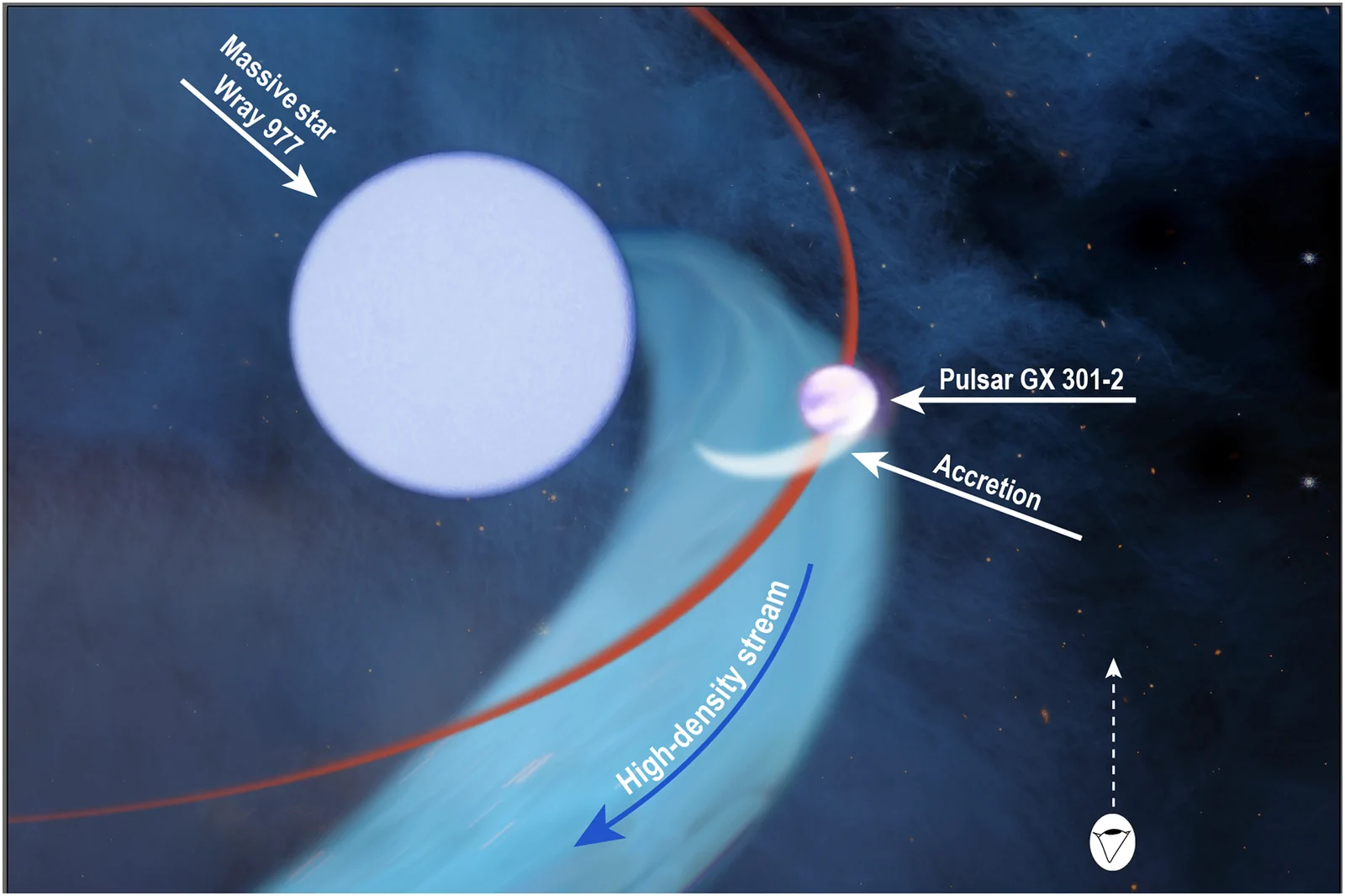
Illustration of the GX 301–2 system. The pulsar is near periastron and embedded in the high-density stream flowing from the massive star Wray 977. The image is meant as a visual aid to understand the hypothesized conditions in this observation and is not to scale. Image credit: NASA GSFC/J. Friedlander.

On 1 February 2025, XRISM observed GX 301–2 for ∼100 ks, with 50-ks net exposure. The observation caught the end of the preperiastron flare. An illustration of the system in the state that it was observed is shown in Fig. 1. During the observation, the pulsar is thought to be exiting the high-density stream. We used HEASoft version 6.35.1 to analyze this observation. We followed the instructions in the XRISM data reduction guide (https://heasarc.gsfc.nasa.gov/docs/xrism/analysis/abc_guide/xrism_abc.html), including exclusion of pixel 27. We analyzed the resulting Resolve spectra using Xspec version 12.15.0f.

### Data analysis

**Fig. 2.**
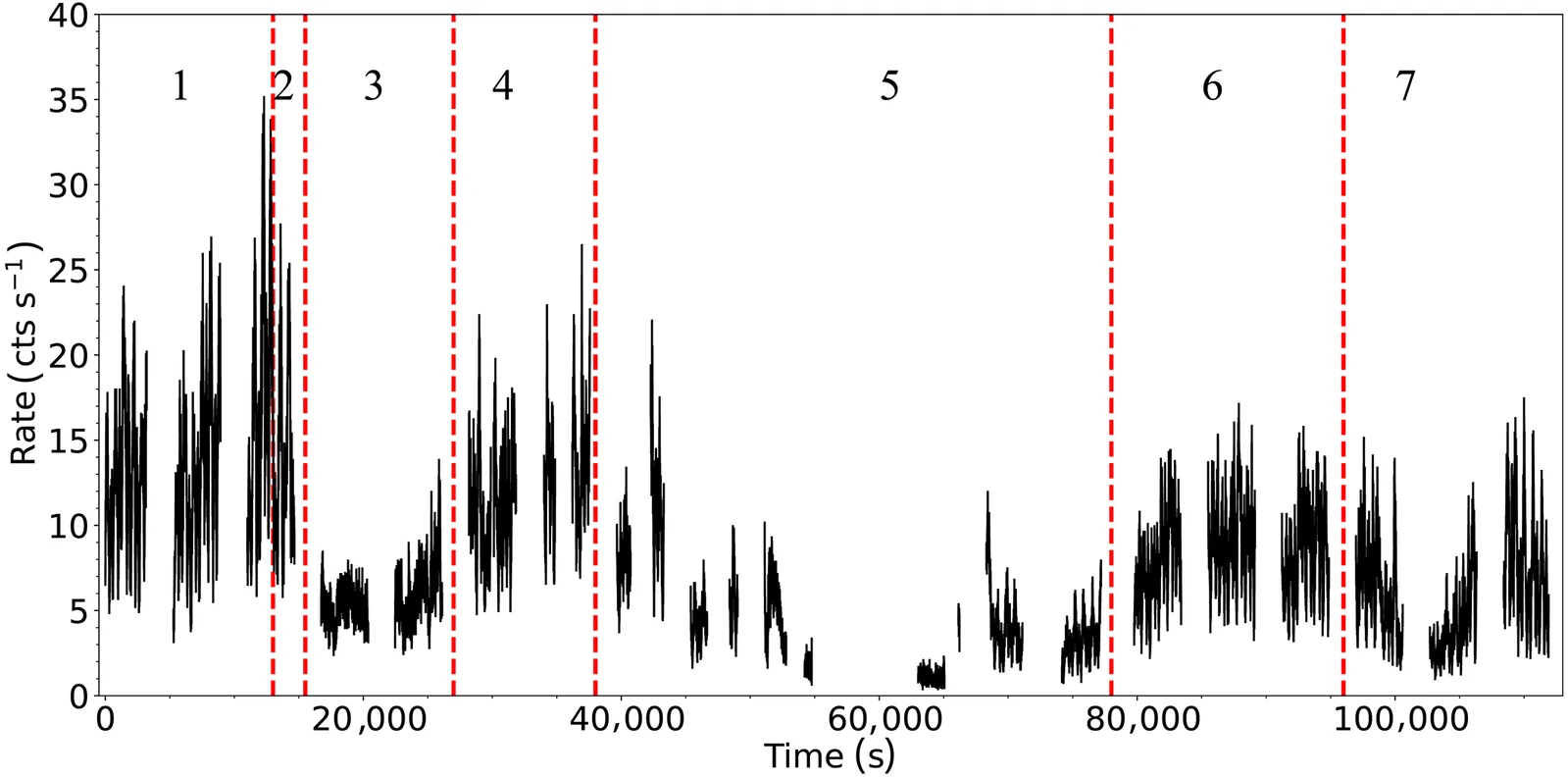
The light curve of the XRISM observation of GX 301–2 in 16-s bins in the 2- to 20-keV band. S1: Absorption. The first 13 ks in the observations. S2: No detectable absorption; ∼2 ks in total. S3: Emission. Broad emission lines are present. S4: Mixed absorption and emission. S5: Emission similar to S3. S6: Mixed absorption and emission. S7: Emission and marginally detectable absorption. The flux decays slowly.

We divide the observation into segments based on the count rate and the presence of absorption and emission lines in the 6.5- to 7-keV range. Figure 2 shows the light curve of the observation and the segments. The segmentation is described in detail in Materials and Methods. In the paragraphs below, all exposure times are net except where indicated.

**Fig. 3.**
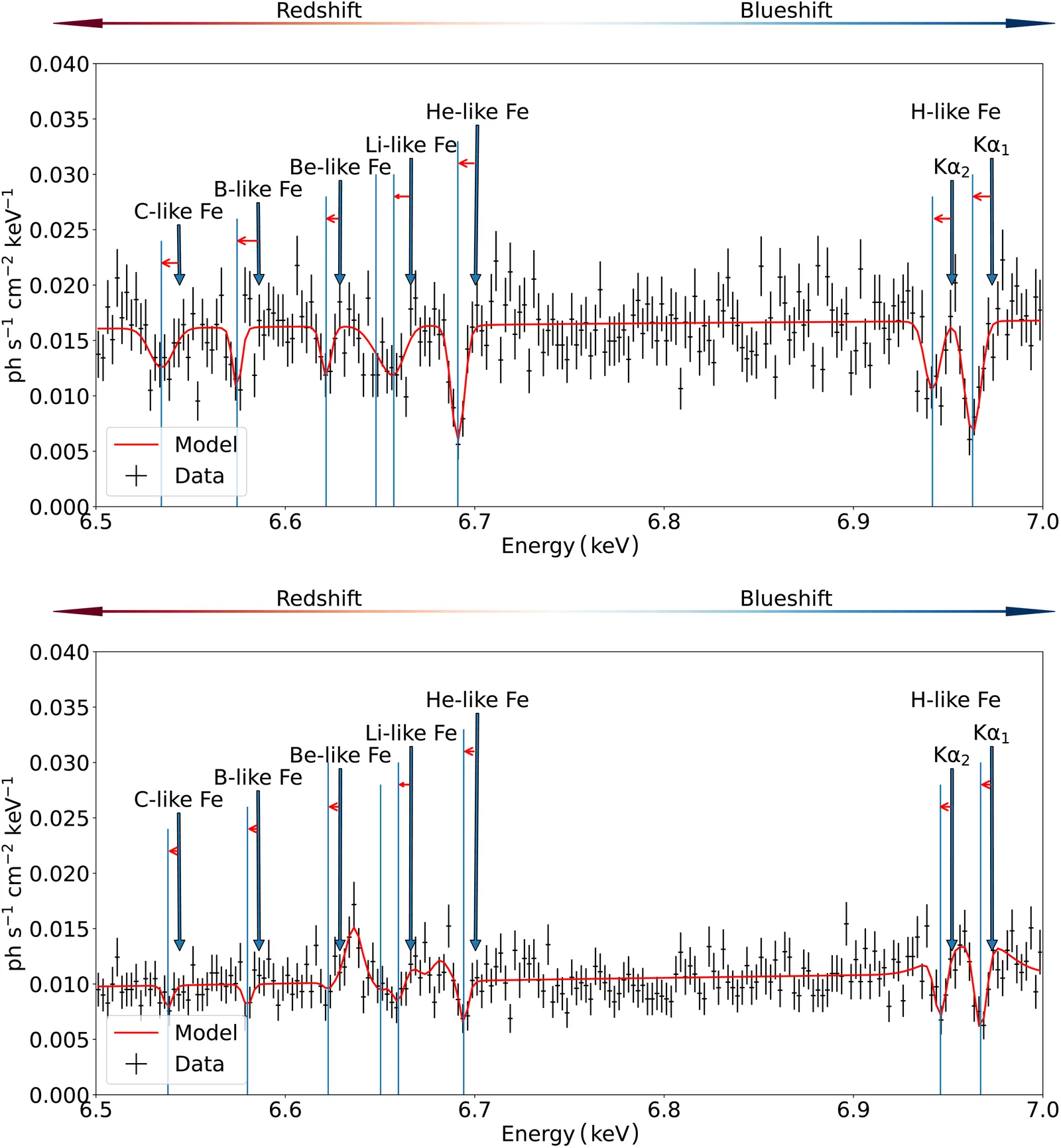
Spectra of GX 301–2 during S1 and S6, where absorption is present and most discernible. Blue arrows show the rest energies, blue lines show the measured energies, and the red arrows show the velocity redshift on each line. Top: S1 spectrum. Redshifted iron absorption lines are visible. Bottom: S6 spectrum. Both emission lines and absorption lines are present. The absorption lines are still redshifted, but substantially less than in S1.

**Fig. 4.**
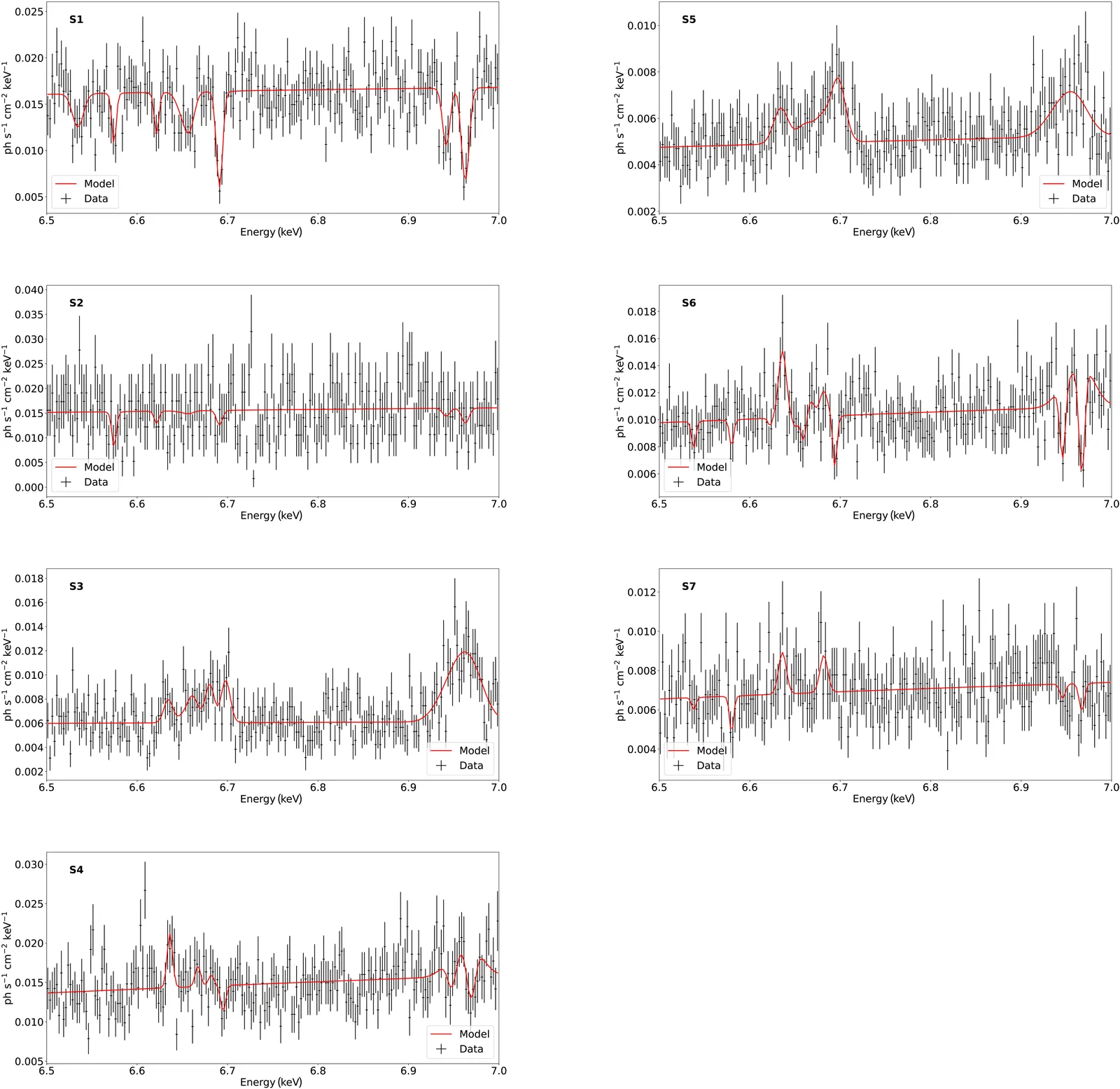
Spectra from all seven segments. S1: Both He-like and H-like iron absorption lines are visible in the spectrum, redshifted by v≈430 km s^−1^. S2: The continuum is similar to S1, but absorption features are absent. S3: Wide emission lines from He-like and H-like iron are visible in the spectrum. S4: Absorption and emission lines are present, with the absorption notably fainter than S1. S5: Clear emission comparable to S3. S6: Both emission lines and absorption lines are present. S7: Absorption lines are faint or undetectable, whereas the emission remains present.

Segment 1 (S1) encompasses the first 13 ks of the observation, with a net exposure time of 8.8 ks. The spectrum contains prominent iron absorption lines, which are redshifted with a velocity of several hundred kilometers per second. The velocity of the most prominent lines, H-like and He-like iron, for example, is ∼430 km s^−1^. The H-like and He-like absorption lines are marginally broadened (∼100 km s^−1^), whereas some lower charge states show a slightly larger broadening but with much higher uncertainty. The H-like and He-like lines are detected with a significance higher than 5σ, implying a definitive detection. The lower charge states are detected individually with a significance of >3σ for the Li-like lines (as a mutual detection), and 2σ to 3σ for lower charge states, implying a marginal detection that is supported by the presence of the H-like and He-like lines. A more detailed analysis of detection significance can be found in Materials and Methods. A detailed list of lines and their velocities and equivalent widths is listed in Table 1. The spectrum and model are shown in Fig. 3 (top). The spectrum of Segment 2 (S2; 1.7 ks; Fig. 4) contains no detectable absorption lines, except a possible detection of B-like iron with a low significance of ∼1σ. This is likely a spurious detection.

**Table 1.** Velocity shifts, broadening, and equivalent width in all detected absorption lines between 6.5 and 7 keV during S1 and S2. Equivalent width upper limits are given when a line is not detected. Velocity shift and broadening are determined by the best fit in S1. All uncertainties are statistical and on the 1σ confidence level. Reference energies are taken from (*38*) and (*51*). The equivalent widths are calculated by definition assuming a constant continuum.

Fe line $E_{\text{ref}}$ (keV)	*v* (km s^−1^)	$\sigma_{v}$ (km s^−1^)	S1 EW (eV)	S2 EW (eV)
XXVI (H-like) Lyα_2_ - 6.9732	$443_{-26}^{+24}$	$137_{-21}^{+24}$	$6.4_{-0.9}^{+1.0}$	≤3.6
XXVI (H-like) Lyα_2_* - 6.9520	$443_{-26}^{+24}$	$137_{-21}^{+24}$	3.8±0.6	≤2.0
XXV (He-like) - 6.7004	$419_{-23}^{+25}$	103±29	5.7±1.0	≤3.3
XXIV (Li-like) - 6.6622	$329_{-62}^{+60}$	$223_{-70}^{+117}$	$3.7_{-1.4}^{+1.6}$	≤2.8
XXIII (Be-like) - 6.6288	$297_{-93}^{+83}$	<130	$1.8_{-0.9}^{+1.2}$	≤2.7
XXII (B-like) - 6.5861	$527_{-41}^{+77}$	<100	$2.1_{-0.9}^{+1.0}$	$3.3_{-1.5}^{+1.7}$
XXI (C-like) - 6.5442	$443_{-422}^{+135}$	$248_{-68}^{+108}$	$3.6_{-1.7}^{+2.9}$	≤0.8

* Fit together with Lyα_2_.

In Segment 3 (S3; 7.4 ks; Fig. 4), ∼4 ks after the absorption lines disappear, the flux is reduced by a factor of ∼2 to 3, and emission lines appear in the spectrum (significance, >5σ). The emission lines are broad ($\sigma_{v}=202_{-41}^{+48}\text{km}s^{-1}$ for the He-like emission and $\sigma_{v}=655_{-120}^{+106}\text{km}s^{-1}$ for the H-like emission) and show a lower redshift ($111_{-64}^{+52}$ km s^−1^ for He like and $272_{-82}^{+114}$ km s^−1^ for H like).

In Segment 4 (S4; 5.6 ks; Fig. 4), absorption lines are again visible in the spectrum (significance ∼3σ). Emission lines are also visible, especially the He-like forbidden line. The absorption lines in this segment have a considerably lower redshift of $213_{-104}^{+79}$ km s^−1^.

In Segment 5 (S5; 14.1 ks; Fig. 4), there are again emission lines in the spectrum similar to S3 (significance >5σ). The line widths are statistically consistent with those in S3.

The flux during Segment 6 (S6; 10.9 ks) is higher than the flux during S3 and most of S5 but lower than the flux during S1, S2, and S4. The S6 spectrum (Fig. 3) shows both emission and absorption. The absorption lines in S6 are redshifted with a lower velocity than S1, statistically consistent with S4. The He-like and H-like absorption lines are jointly detected at a higher than 5σ significance, other absorption lines are marginal at ∼1 to 2σ.

Segment 7 (S7; 10.7 ks; Fig. 4) starts ∼20 ks after the start of S6. In S7, the absorption lines in the spectrum are no longer detected with any significance, reminiscent of S2, while the flux gradually decreases. Toward the end of S7, there are increases in the flux, but the spectrum remains statistically consistent with earlier parts of S7. S7 is a notably longer segment than S2 without substantial spectral changes, and the observation ends before there is a clear transition to another state. The measured velocities and equivalent widths of lines in S6 and S7 appear in Table 2.

**Table 2.** Velocity shifts, broadening, and equivalent width in all detected absorption lines between 6.5 and 7 keV during S6 and S7. Equivalent width upper limits are given when a line is not detected. Velocity shift and broadening are determined by the best fit in S6. All uncertainties are statistical and on the 1σ confidence level. Reference energies are taken from (*38*) and (*51*). The equivalent widths are calculated by definition assuming a constant continuum.

Fe line $E_{\text{ref}}$ (keV)	*v* (km s^−1^)	$\sigma_{v}$ (km s^−1^)	S6 EW (eV)	S7 EW (eV)
XXVI (H-like) Lyα_2_ - 6.9732	265±20	$83_{-19}^{+16}$	6.0±2.0	$1.5_{-1.2}^{+1.4}$
XXVI (H-like) Lyα_2_* - 6.9520	265±20	$83_{-19}^{+16}$	$3.9_{-1.2}^{+1.4}$	$0.9_{-0.7}^{+0.8}$
XXV (He-like) - 6.7004	$280_{-47}^{+46}$	<114	$2.1_{-1.1}^{+1.2}$	≤0.9
XXIV (Li-like) - 6.6622	$292_{-104}^{+108}$	<213	$0.9_{-0.6}^{+0.6}$	≤0.5
XXIII (Be-like)† - 6.6288	$280_{-47}^{+46}$	<114	≤1.1	≤0.4
XXII (B-like)† - 6.5861	$280_{-47}^{+46}$	<114	$1.2_{-0.8}^{+1.1}$	$1.6_{0.6}^{+1.4}$
XXI (C-like)† - 6.5442	$280_{-47}^{+46}$	<114	$1.2_{-0.7}^{+1.1}$	≤1.5

* Fit together with Lyα_2_.

† Fit using the same velocity and broadening of Fe XXV, due to low confidence.

The absorption lines in S1, S4, and S6 are from highly ionized iron, which requires a high ionization parameter (*32*). For a high-density wind, such as in GX 301–2, this implies relatively close proximity to the pulsar. These lines may therefore trace inflowing matter actively being accreted by the compact object. As the absorption ceases, the luminosity rapidly decreases to a level similar to the off-states observed in the past using XMM-Newton (*28*), a factor of ∼2 to 4 times lower luminosity relative to segments with observed absorption. The presence of emission lines and the statistical quality of the spectrum in S4 prevent us from placing meaningful constraints on the absorption lines. Therefore, the analysis in this work will focus on S1 and S6.

### Testing whether the absorber is being accreted

We test our interpretation by analyzing the properties of the absorbing medium during S1 by fitting the spectra using the photoionization code xstar (*32*) with a spectral energy distribution appropriate for GX 301–2. We find the spectrum is best described by a medium with electron number density $n∼10^{12}{\text{cm}}^{-3}$, an ionization parameter $\text{log}\xi =3.2_{-0.2}^{+0.1}$ ($\xi \equiv \frac{L}{{\mathrm{nr}}^{2}}$, in cgs units), and a column density of $N_{H}∼3\times 10^{22}$ cm^−2^. Using these measurements, we estimate the absorber to be at a distance of $r∼7\times 10^{10}$ cm from the x-ray source (the neutron star) and have a size of $d∼3\times 10^{10}$ cm. The absorber is therefore located fully within the accretion radius of the neutron star ($r_{a}=2\mathrm{GM}/v_{\text{wind}}^{2}\geq 5\times 10^{11}\;\text{cm}$). As a consistency check, we can use the size and distance to estimate the mass accretion rate of $\dot{M}\approx 2.1\times 10^{16}\;g\;s^{-1}\approx 3.2\times 10^{-10}\;M_{⊙}\;{\text{year}}^{-1}$ during this event. We then derive an expected bolometric luminosity from this accretion rate of $L∼2\times 10^{36}\;\text{to}\;7\times 10^{36}\;\text{erg }s^{-1}$, which is comparable to the luminosity $L=6.3\times 10^{36}$ erg s^−1^ observed during S1. The expected luminosity is not required to account for the entire derived luminosity, as a baseline luminosity exists outside of these flare, but it provides an order of magnitude comparison, as the flaring luminosity is two to four times the baseline luminosity in this observation. The full analysis is discussed further in Materials and Methods.

All these aspects of the observation thus strongly support the hypothesis that the absorption originates in matter being actively accreted by the neutron star. The redshift indicates inflow, the absorber is positioned within the accretion radius, the luminosity resulting from this accretion is in line with that observed, and, once absorption is no longer detected, the luminosity decreases rapidly.

Alternative explanations for the observed redshifted absorption are physically inconsistent. Such explanations include a clump of wind reversing direction and falling back toward the star (*33*) or spontaneous shocks in the wind. Considering the slow velocity of the GX 301–2 wind [∼300 km s^−1^; (*23*)], shocks bringing the temperature to >1 × 10^7^ K are difficult to explain and not supported observationally in the system. Clumps reversing direction may be possible, but the observed velocity of ∼400 km s^−1^ is inconsistent with a distance $>0.5R_{*}$ required for material to be on the line of sight. Even if the velocity could be explained, the ionization parameter still requires proximity to the x-ray source, unless the clump density is substantially lower than the density of the wind, which is physically unlikely. In light of the implausibility of alternative explanations, we adopt the hypothesis that the observed absorption represents matter actively being accreted onto the neutron star.

According to a model for the flares in GX 301–2 (*24*), which is supported by multiple observational studies (*23*, *29*, *30*), the preperiastron flare is caused by the neutron star overtaking and crossing a high-density stream. We therefore interpret the observed inflow as a clump of matter pulled from the stream by the passage of the neutron star. This marks the first-ever direct observation of active wind accretion in a HMXB.

The emission lines seen in S3 and S5 have lower redshift and higher broadening, both of which suggest a different origin for the emission and absorption lines. Their persistence without the presence of absorption lines supports this interpretation. The emission lines could potentially originate from a sparser gas, potentially the stellar wind constantly flowing out from the companion. In this case, the emitting plasma would be found farther away from the pulsar, to maintain a similar ionization parameter for the presence of highly ionized iron. The emitting material being a sparser stellar wind could explain the higher broadening and lower redshift. It is possible that this material is getting accreted constantly by the pulsar, not in any specific event, supporting the ongoing lower-flux emission, but it could also simply be the photoexcited stellar wind. We find that we cannot distinguish the cases in this observation, and, because the emitting material is unlikely to be related to the absorbing material, we do not analyze it further in this work.

The first event (S1 and S2) and the last event (S6 and S7) are substantially different in their inflow velocity and luminosity. The observed luminosity in S6 is lower by a factor of ∼2 compared with that in S1, whereas the inflow velocity is consistent with 0, meaning the redshift velocity is approximately equal to the orbital velocity of the pulsar. Additionally, despite the absorption having a comparable equivalent width, the light-curve evolution is different. In S1, the flux increases until there is an abrupt transition from a spectrum showing absorption to one showing no absorption (see Fig. 4). At that point, the flux decays over ∼2 to 4 ks to considerably lower levels. In S7, after reaching a peak, the light curve plateaus for ∼10 ks and then decays gradually over another ∼10 ks while showing reduced or nonexistent absorption. The flux then increases before the end of the observation, but the observed spectrum does not change and absorption lines are still absent.

S4 shows fainter absorption compared with S1 and S6 and a comparable redshift to S6. We can reasonably conclude that it is an additional accretion event, but the lower absorption and limited statistical certainty preclude more meaningful statements.

### Near-direct flow versus disk-like in-spiral

We speculate that the differences between the first and last accretion events stem from a difference in the angular momentum of the inflow. Specifically, the radial velocity being consistent with 0, the light-curve plateau, and the slow decay of the light curve during S7 would be simpler to explain for an in-spiral than for a near-radial flow. The free-fall time from a radius of ∼1 × 10^10^ cm (the estimated absorber distance) onto a $∼2M_{⊙}$ compact object is ∼1 ks. For near-radial accretion, one thus expects a rapid reduction in flux once the absorber reaches small radii and is fully ionized, as seen in S2. Conversely, a high-angular-momentum inflow will form a disk-like structure, temporarily inhibiting accretion and prolonging the event length, as seen in S7. A disk-like structure is also expected to show very low radial inflow velocity, as seen in S6. GX 301–2 was previously hypothesized to create short-lived accretion disks (*26*, *34*, *35*). Transient accretion disks were proposed as an explanation for spin-up events (*26*, *34*), and a follow-up study (*35*) described both wind and disk accretion in an attempt to find characteristics of different accretion modes in GX 301–2.

Xu and Stone (*36*) examined unstable flow in wind-fed x-ray binaries and formulated the conditions for stable flow, turbulent flow, and disk formation in HMXB accretion. They point to the transverse density gradient as one of the main predictors for the formation of disk-like structures. For most of its orbit, GX 301–2 falls into the “turbulent flow” category, where intermittent accretion breaks are expected, but without disk formation. However, this model, like most wind accretion models, assumes matter is accreted from the spherical stellar wind. In GX 301–2, flares are caused by the neutron star crossing a high-density stream, leading to a sudden and sharp increase in density. This increase by a factor of ∼20 to 30 in under a day is easily sufficient to produce turbulent disk-like structures at the start and end of GX 301–2 flares. Because the sign of the disk angular momentum depends on the sign of the density gradient (*36*), we expect alternating prograde and retrograde disks in each stream crossing.

Koh *et al*. (*26*) raised the possibility of disks in GX 301–2 but emphasized that, if disks form, then they must normally alternate between prograde and retrograde with timescales ∼4 days. This timescale is approximately the length of the flare, meaning the time that it takes the neutron star to cross the high-density stream.

**Fig. 5.**
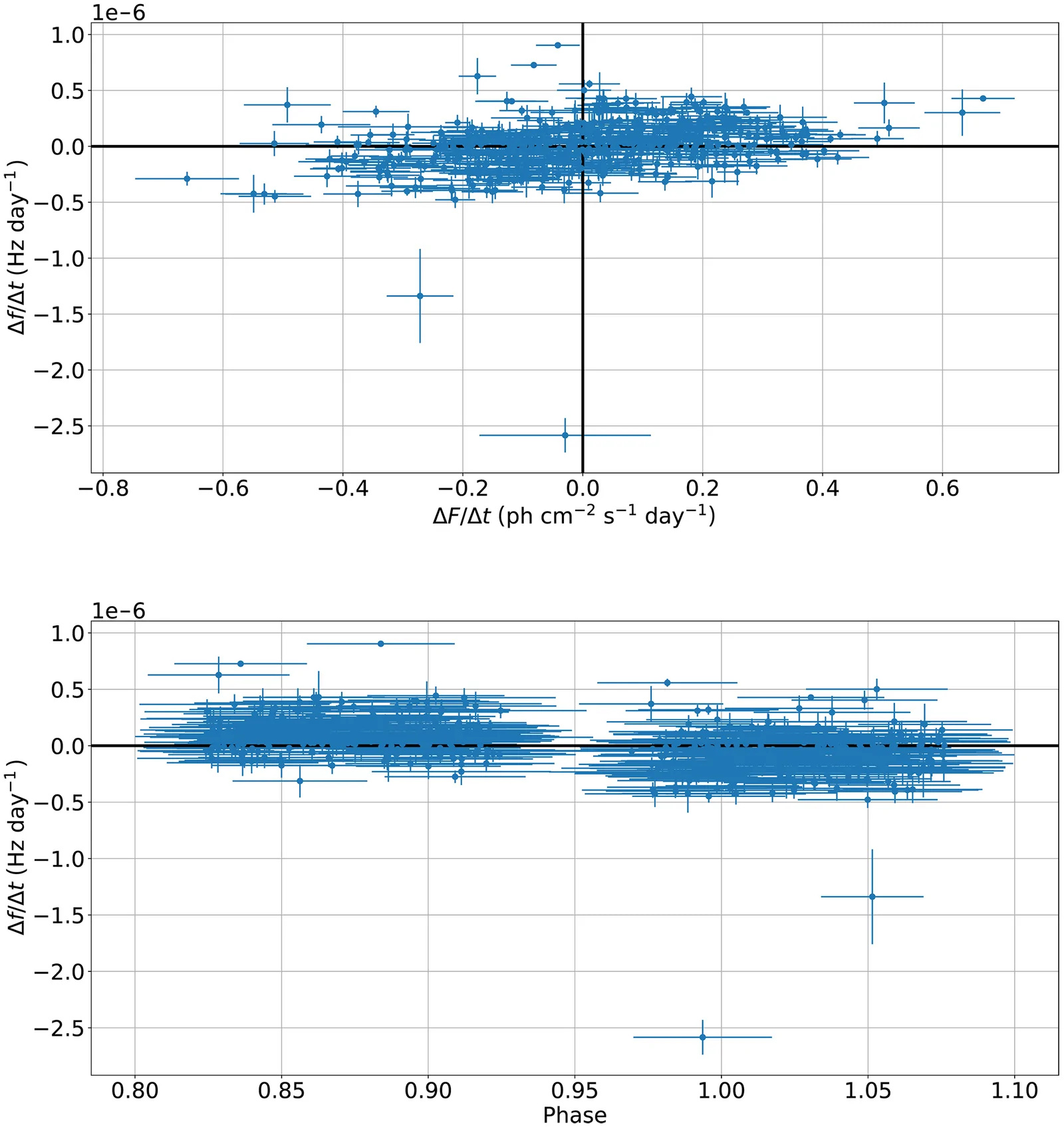
The change in pulse period plotted against change of observed flux and phase. (**Top**) The change in the pulse period of the pulsar GX 301–2 plotted against the change in observed flux in the 12- to 50-keV range. (**Bottom**) The change in the pulse period of the pulsar GX 301–2 plotted against the phase. The correlation between a change in flux and a change in pulse period is readily apparent. Also apparent is the tendency for entry into the stream to increase the frequency, whereas exiting the stream decreases it.

We test our hypothesis by examining the long-term light curve and pulse profile history of GX 301–2 as measured by the Fermi/Gamma Burst Monitor (GBM). The time derivative of the pulse period evolution shows a weak spearman rank correlation with the time derivative of the 15- to 50-keV flux ($\rho_{s}∼0.2,P<0.01$). When we select for the flare start and end phases, the correlation increases to $\rho_{s}∼0.4,P<0.01$. If we also select for the events with the greatest change in flux or only statistically significant frequency changes, then it increases further to $\rho_{s}∼0.5$. Additionally, we test whether frequency increases are more common during flare start and frequency decreases during the end of a flare. We calculate an odds ratio of $6.0_{-1.1}^{+1.2}$ implying a notable association between the start and end of the preperiastron flare and the sign of the frequency change. A full analysis is shown in Materials and Methods. The data points during the beginning and end of flare are shown in Fig. 5. The figure shows both the flux-frequency relation and the phase-frequency relation. The implication is that the angular momentum tends to increase when flares start and decrease when they end. This fits what is expected from prograde disks followed by retrograde disks.

Alternative explanations for this result are possible. First, the correlation could be driven by the secular evolution of the spin period of GX 301–2. We test this by comparing our calculated odds ratio to the odds ratio of an arbitrary choice of “start” and “end” phases. An arbitrary choice in the interval [0.2,0.8] has an odds ratio of ∼1.5 at most, implying that a small part of the effect may be, in part, explained by secular evolution, but the effect of the flare is at least ∼4 times stronger. We therefore reject the secular evolution as a main driver of our result. The result could instead indicate a more complex scenario where the flow becomes more turbulent, but in-spirals do not form. Additionally, there are inherent limitations in the data. The frequency estimates of GX 301–2 are done using a template pulse profile. Changes in the pulse profile could influence the frequency measurements, and it is possible for the pulse profile itself to be coupled to flux changes regardless of the presence of disk-like structures.

Despite these limitations, we argue that the evidence shown here favors a hypothesis of multiple accretion forms in GX 301–2. Most of the time, GX 301–2 accretes matter with low angular momentum that forms a turbulent, but mostly radial, inflow (such as during S1). However, at the beginning and end of the preperiastron flare (such as in S6), as the neutron star enters and exits the stream, GX 301–2 may have disk-like in-spirals. The beginning of the flare was not captured in this observation and a follow-up observation showing the start of a preperiastron flare may serve to validate or reject this hypothesis.

GX 301–2 is thus an ideal system to study wind-fed accretion in HMXBs. It possibly displays both mostly radial inflow as well as turbulent disk-like structure formation. If true, both inflow modes are directly observable using XRISM/Resolve and the science of wind accretion can be greatly enhanced by additional observations studying the redshifted absorption lines of highly ionized iron in this and similar systems. Other systems that may lend themselves to such studies include Vela X-1 [see (*37*)] and 4U 1700-37.

## MATERIALS AND METHODS

### Data analysis

The XRISM observation of GX 301–2 shows large variability in count rate. When dividing the observation into segments, we first divide on the basis of the count rate and light-curve behavior. We separate at the observation gaps based on substantial changes in the count rate. This initial segmentation resulted in five segments, the first of which is from *t* = 0 to *t* = 15,000 s (S1 and S2). We then create a more refined subdivision using a sliding window of 1500 s, shifted by 500 s in each step. In each step, we examined the resulting spectrum and analyzed the absorption present in it. Around the *t* = 13,000-s mark (the end of S1), the absorption lines begin to weaken until they disappear completely once the time window start point is at 13 ks; we thus define S2 as *t* = 13,000 s to *t* = 15,000 s. A similar result occurs for the final segment around the 95-ks point. We therefore subdivided the first and last segments according to their spectral characteristics. It is possible to subdivide S5 and S7 further based on count rate, but the resulting spectra do not differ from each other and from their combined forms, and this only results in degrading the statistical significance of the data. We therefore opted not to split S5 or S7 further.

We fit the data in all spectra using an absorbed power-law model with emission or absorption lines added as needed. All data are fit only in the 6.5- to 7-keV range. When fitting a narrow energy band with an absorbed power law, there is degeneracy between the column density and power-law index. To allow a faster convergence of our models, we fix the power-law index to 1, a typical value for this system (*24*, *29*). The power-law index and column density derived from this narrow-band model do not reflect real physical conditions and are merely used to establish a continuum flux being absorbed. Absorption lines are described using a Voigt profile as their velocity dispersion (typically, $\sigma_{v}∼1\;\text{to}\;2$ eV or 50 to 100 km/s) is low enough that the natural width is not negligible. The emission lines are broad ($\sigma_{v}∼4.5$ eV or 225 km/s for He-like and ∼12 eV or 550 km/s for H-like lines) and are fit with a Gaussian profile. The width difference between the He-like and H-like lines is statistically significant and may reflect a different distance from the source. We do not explore this further in this study. When using Voigt profiles, we fix the natural line width using measured values (*38*). Because they come from the same ion, the H-like Lyα_1_ and Lyα_2_ share the same redshift and width, and the ratio of depths set by their oscillator strengths. The Li-like Kα lines *q* and *r* (*39*) are similarly tied. Because the data are described in counts per channel, we fit the data using the Cash statistic (*40*).

We tested the significance of our detections of absorption and emission features by comparing our best fit models to models without those features, including in Month Carlo simulations (*41*). In S1, a fit without H-like and He-like absorption features increases the fit statistic by 140. The redshift of the H-like and He-like absorption lines was tied to reduce the number of free parameters and allowed their significance to be tested jointly, thus implying a joint significance much higher than 5σ. Individually, each line is still detected at a significance higher than 5σ in S1. Because of the presence of three lines from two charge states at the same redshift, there is enough prior information in this case and the line detection cannot be considered blind, and we therefore consider the H-like and He-like absorption lines to be definitively detected. Lines from lower charge states (Li like to C like) are detected at a much lower marginal significance. The Li-like lines are detected at a significance of >3σ based on Monte Carlo simulations, whereas lower charge states are detected at 2σ to 3σ. We consider these detections in S1 as likely because their velocities are similar to the H-like and He-like lines and because the presence of adjacent lower charge states is plausible in a photoionized plasma with a moderate distribution of ionization parameters. Even if some or all of the lower charge state lines are spurious detections, our analysis does not rely on them, and the conclusions of this paper remain the same.

During S2, the only marginally significant absorption line detected is from B-like iron. Based on simulations, this is an ∼1σ detection. This is likely a spurious result because lines from neighboring charge states that would be expected to be comparably detectable were not found.

During S6, the only absorption lines detected with meaningful significance are He-like and H-like iron. The model involves emission and is thus more complex. However, the inclusion of absorption features still results in an improvement in the fit statistic of ∼50, implying a joint significance exceeding 5σ. Individually, the He-like line is detected at ∼3σ and the H-like complex at >4σ.

S6 features both emission and absorption lines. When fitting the He-like emission complex in S6, we assume the broadening and redshift velocities of all He-like emission lines are equal. The resulting velocities are statistically consistent with those obtained using the same method in S3. When fitting the H-like emission lines, their overlap with the absorption lines prevents the direct fitting of their velocities. We therefore only allow the amplitude of the Lyα_1_$\alpha_{1}$ line to change while assuming that their redshift and broadening are identical to those found in S3. We then fit the absorption lines as described in S1. Using this method, we obtain the absorption lines’ equivalent width and positions in Table 2. Using the xstar table models to fit the spectrum of S6 yields statistically consistent column density and ionization parameter results to those obtained for S1, but with greater confidence margins.

### Estimating absorber parameters

To determine the absolute velocity of the absorber relative to the neutron star, we must account for the following four velocity components: the neutron star relative to the B hypergiant, the velocity of the GX 301–2 system relative to the solar system, the velocity of Earth around the sun, and the velocity of XRISM around the Earth. The velocity of XRISM is <10 km s^−1^ and can be neglected. The radial velocity contribution stemming from the motion of the Earth around the sun is $v_{\text{earth}}\approx 20$ km s^−1^. The radial velocity of the GX 301–2 system relative to the solar system is $v_{\text{system}}=-30$ km s^−1^ (*23*). The largest velocity component is the radial velocity of the neutron star, which is estimated from the phase and orbital parameters, and thus depends on our choice of those parameters. Multiple works measured the orbital period and rate of change in GX 301–2 (*25*–*27*), and the differences in their determinations result in up to ∼0.15 in phase uncertainty for our observation. If we adopt the values of Doroshenko *et al*. (*27*) (*P* = 41.506 days, $\dot{P}=-3.7\times 10^{-6}$ s s^−1^, $T_{\text{PA}}=43906.06$ MJD), then we get ϕ≈0.1 and $v_{r,\text{NS}}\approx 236$ km s^−1^. If we adopt the values of Koh *et al*. (*26*) (*P* = 41.488 days, $T_{\text{PA}}=48802.85$ MJD, constant period), then we get ϕ≈0.95 and $v_{r,\text{NS}}\approx 24$ km s^−1^. The highest neutron star velocity is found when we adopt the period and epoch from Koh *et al*. (*26*) and use the orbital period decay rate of $\dot{P}=-2\times 10^{-6}$ s s^−1^ found by Manikantan *et al*. (*42*), yielding ϕ≈0.03 and $v_{r,\text{NS}}\approx 277$ km s^−1^. After correcting for all velocity components $\left(v_{\text{absorber}}=v_{\text{redshift}}-v_{r,\text{NS}}-v_{\text{earth}}-v_{\text{system}}\right)$, the velocity of the absorber relative to the pulsar is greater than 150 km s^−1^ in all described cases. This implies substantial movement of the absorber toward the neutron star. This makes this observation the first in which x-ray absorption is detected from an ionized inflow rather than an outflow. See Table 3 for a full list.

**Table 3.** Absorber velocity relative to pulsar under different period assumptions.

Source	$T_{\text{PA}}$ (MJD)	*P* (days)	$\dot{P}$ (s s^−1^)	ϕ	$v_{\text{NS}}$ (km s^−1^)	$v_{\text{absorber}}$ (km s^−1^)
(*26*)	48802.85	41.488	0	0.95	24	∼400−430
(*27*)	43906.06	41.506	$-3.7\times 10^{-6}$	0.1	236	∼190−220
(*26*, *42*)	48802.85	41.488	$-2\times 10^{-6}$	0.03	277	∼150−180

In this work, we adopt the higher velocity value predicted by combining the orbital parameters of Koh *et al*. (*26*) with the decay rate found by Manikantan *et al*. (*42*). We find that this phasing is consistent with the long-term light curve of GX 301–2 in the epoch of our XRISM observation. This choice does not fundamentally affect the analysis or conclusions presented here.

**Fig. 6.**
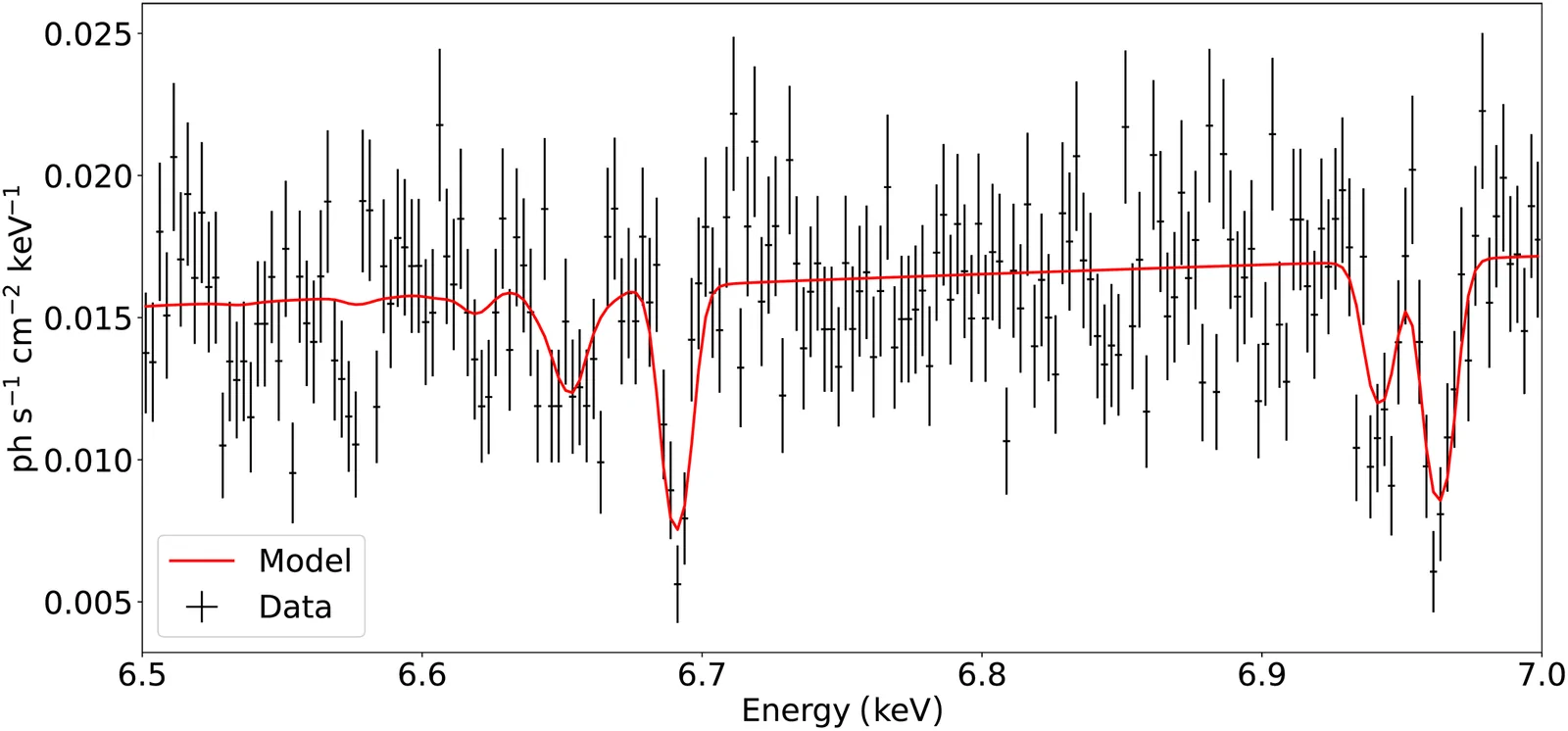
The spectrum of S1 with the table model with density of $n=10^{12}$ cm^−3^. xstar successfully recreates the most prominent absorption features in the spectrum.

We calculated sets of table models with the photoionization code xstar (*32*) for a spectral energy distribution appropriate for GX 301–2. In these models, we vary the density from 10^11^ to 10^13^ cm^−3^ while assuming that the density is constant across the absorbing medium. This allows us to estimate the geometrical size of the absorbing medium along the line of sight. The best fit for the model at 10^12^ cm^−3^ is shown in Fig. 6. We do not explore lower densities as they result in an absorber size far too large compared with the derived absorber position, as discussed below. Much higher densities would be difficult to explain in the context of the density of the surrounding material.

We use values provided by Leahy *et al*. (*24*) to estimate the density of the wind and stream in the vicinity of the neutron star. In their work, the terminal velocity is between 350 and 620 km s^−1^ and the mass loss rate of the wind is between $8.9\times 10^{-7}M_{⊙}{\text{year}}^{-1}$ and $2.8\times 10^{-5}M_{⊙}{\text{year}}^{-1}$, depending on the radius of the stellar companion and the inclination of the system. We can calculate the density of the wind and stream using the continuity equation

$$n=\frac{\dot{M}}{4\pi r_{d}^{2}v\mu m_{H}}$$ (1)

where $\dot{M}$ is the mass loss rate, $r_{d}$ is the distance from the companion, *v* is the velocity, and we take $m_{H}=1.67\times 10^{-24}$ g. We calculate the velocity from a simple wind profile with $v=v_{\infty }{\left(1-R_{*}/r_{d}\right)}^{\beta }$. We assume here β = 1, as in Leahy *et al*. (*24*). Kaper *et al*. (*23*) found β = 1.75. Adopting this would slightly increase the density estimate but would not affect our conclusions.

Using the aforementioned values and the orbital parameters described by Koh *et al*. (*26*) [$e=0.462;a_{x}\text{sin}\left(i\right)=370.0$ lt-s] and the mass loss rate described by Leahy *et al*. (*24*), we calculate the density of the wind and the stream. The exact mass loss rate is not known, and so we calculate the density for the entire sample set supplied by Leahy *et al*. (Table 1) (*24*). The wind density in the vicinity of the neutron star (not accounting for any effects from the neutron star) is between 3 × 10^9^ and 6 × 10^10^ cm^−3^. The stream density, based on the density increase parameter (*24*), is between 6 × 10^10^ and 2 × 10^12^ cm^−3^ in agreement with our xstar models that indicate densities above 10^11^ cm^−3^. Because this observation was performed during the preperiastron flare, we expect accretion from the high-density stream. Tidal effects on a clump of matter during accretion will further increase the density, making a density of $n∼10^{12}$ cm^−3^ or even $n∼10^{13}$ cm^−3^ likely.

We can use the density and luminosity to estimate the distance between the source and absorber. Each model has a different log(ξ) in the best fit. The ionization parameters are $4.1_{-0.3}^{+0.8}$, $3.2_{-0.2}^{+0.1}$, and $3.1_{-0.1}^{+0.05}$ for $n=10^{11}$, 10^12^, and 10^13^ cm^−3^, respectively. This describes the ionization parameter at the front of the absorber. We infer the mean unabsorbed flux from the best-fit absorbed power-law model of the 3- to 12-keV spectrum during S1. The inferred unabsorbed flux during S1 in the 13.6- to 13.6-keV range is 3.3 × 10^−9^ erg s^−1^. We selected this specific spectral range for consistency with the xstar definition of the ionization parameter. We adopt the Gaia distance of 3.99±0.25 kpc (*43*) and get an average luminosity during S1 of $L=6.3\pm 0.8\times 10^{36}$ erg s^−1^. Coupled with the density and ionization parameters shown above, we can estimate the distance of the absorber from the x-ray source (the pulsar)

$$r=\sqrt{\frac{L}{n\xi }}∼2-7\times 10^{10}\text{cm}$$ (2)

where the closer distance corresponds to the higher density. This is well within the accretion radius $r_{a}=2\mathrm{GM}/v_{\text{wind}}^{2}\geq 5\times 10^{11}\text{cm}$.

We can estimate the size of the absorber using the electron number density and best-fit column density of $N_{H}∼3\times 10^{22}{\text{cm}}^{-2}$, obtaining

$$d=N_{H}/n_{e}∼3\times 10^{10}\text{cm}\left(\frac{10^{12}{\text{cm}}^{-3}}{n_{e}}\right)$$ (3)

We can now estimate the mass inflow rate and the corresponding predicted luminosity, which we can then compare with the observed luminosity to test for consistency. Fulfilling the continuity equation, for spherically symmetrical accretion, the mass accretion rate onto the compact object is equal to the mass passing through a shell of radius *r* around the central object per unit time. For nonspherical geometries, this area is reduced by the respective solid angle Ω. The mass passing the shell per unit time and area is calculated from the particle number density *n* (equivalent to the electron number density), the average particle mass $\mu m_{p}$ and the inflow velocity *v*. The mass inflow rate can be estimated using

$$\dot{M}=\Omega r^{2}\mathrm{vn}\mu m_{p}$$ (4)

We can estimate Ω by assuming the infalling matter to be roughly spherical and using the estimated size and distance of the absorber calculated previously, so that $\Omega ∼\pi {\left[\text{arctan}\left(\frac{d}{2r}\right)\right]}^{2}$. For sufficiently small Ω, we use the leading term in the series expansion of arctan to write

$$\dot{M}=\frac{\pi }{4}d^{2}\mathrm{vn}\mu m_{p}=\frac{\pi }{4}\frac{N_{H}^{2}}{n}v\mu m_{p}$$ (5)

We assume *v* = 150 km s^−1^ (based on the observed redshift and assumed neutron star radial velocity of ∼240 km s^−1^) and μ = 1.2. For $n\geq 10^{12}{\text{cm}}^{-3}$, we get $\frac{d}{2r}<0.25$, and the approximate form in Eq. 5 is accurate to a few percent. In this case, we find $\dot{M}\approx 2.1\times 10^{16}\;g\;s^{-1}n_{12}^{-1}=3.2\times 10^{-10}\;M_{⊙}\;{\text{year}}^{-1}n_{12}^{-1}$, where $n_{12}^{-1}$ is the density in units of 10^12^ cm^−3^. For $n=10^{11}$ cm^−3^, we must use Eq. 4, finding d∼4r and Ω∼1.2, and $\dot{M}\approx 5.6\times 10^{16}\;g\;s^{-1}\approx 9\times 10^{-10}\;M_{⊙}\;{\text{year}}^{-1}$.

The luminosity derived from this accretion rate is (*44*)

$$L=\frac{r_{g}}{2R}\dot{M}c^{2}$$ (6)

where $r_{g}$ is the Schwarzschild radius of the neutron star and is *R* the physical radius of the neutron star. For a neutron star mass of $M=1.85\pm 0.6M_{⊙}$ (*23*) and a neutron star radius of between 10 and 15 km, we get a bolometric luminosity of $L∼6\times 10^{36}\text{to}\;18\times 10^{36}\text{erg}s^{-1}$,$L∼2\times 10^{36}\;\text{to}\;7\times 10^{36}\text{erg }s^{-1}$, and $L∼2\times 10^{35}\text{to}\;7\times 10^{35}\text{erg }s^{-1}$ for $n=10^{11}$, $n=10^{12}$, and $n=10^{13}$ cm^−3^ respectively. The predicted luminosity range of both $n=10^{12}$ cm^−3^ and $n=10^{11}$ cm^−3^ agrees with the observed luminosity during S1 (6.3 × 10^36^ erg s^−1^), implying a possible self-consistent solution with *n* between 10^11^ and 10^12^ cm^−3^. This result does not materially change if we consider the neutron star’s baseline luminosity and require the active accretion event contribution to be ∼4.5 × 10^36^ erg s^−1^.

### Disk-like structures

In most wind-fed systems, accretion is assumed to be a quasi-spherical inflow (*45*) from wind crossing the accretion radius of the compact object. Still, there are cases where accretion is thought to occur through transient disk-like structures. Observations of the HMXB OAO 1657-415 favor the formation of accretion disks in the system (*46*), and some research into GX 301–2 (*26*, *34*), Vela X-1 (*47*), and 4U 0114+650 (*48*) suggests that they may form transient accretion disks. For GX 301–2, disk formation is supported by spin-up periods throughout the system’s lifetime (*34*). Disk formation is assumed to occur when the angular momentum of the accretion flow is large. Karino *et al*. (*49*) use analytical calculations to argue that disk forming most likely occurs in systems with short orbital periods and slowly rotating neutron stars. Systems with a long orbital period may form disks if the mass-loss rate is very high and the wind is very slow. Xu and Stone (*36*) use two- and three-dimensional hydrodynamic simulations to explore the behavior of wind-fed accretion in HMXB. They characterize three accretion regimes primarily based on the transverse gradient of the wind density. A smooth wind allows for steady accretion, while a more variable density creates a turbulent accretion flow and may lead to transient disk-like structures at very high transverse density gradients, i.e., along the orbital path.

As discussed above, we hypothesize that GX 301–2 forms short-lived disk-like structures upon entering and exiting the high-density stream, based on the longer decay time of the light curve in S7, uncharacteristic of near-direct inflow. Structures formed upon leaving the stream should have opposite angular momenta to those formed when entering the stream, due to the inversion of the density gradient (*36*). Although short-lived (∼10 ks), typical accretion events have a high mass accretion rate (10^16^ to 10^17^ gs^−1^, total of ∼10^21^ g) that could change the pulsar spin frequency by ∼0.01 to 0.1% (assuming a tangent velocity of 200 to 500 km s^−1^ at *r* ∼ 7 × 10^10^ cm). Changes of this magnitude should be seen with instruments following the pulsar persistently.

Fermi/GBM has extensive data on the pulsar frequency and observed flux of GX 301–2 since 2009. Data are recorded regularly, typically 1 to 2 days apart, but some measurements are taken up to ∼20 days apart. We calculate the relative time-dependent flux difference, defined as $2\frac{F_{2}-F_{1}}{\left(T_{2}-T_{1}\right)\left(F_{2}+F_{1}\right)}$ [errors were calculated using SE propagation; (*50*)], and select only those data points that occur between phases 0.8 to 0.9 (entering the steam) and 0.9 to 0.1 (exiting the stream). The phase was calculated using the epoch measured by Koh *et al*. (*26*), and the orbital decay calculated by Manikantan *et al*. (*42*). We then calculate the change in pulse frequency for these data points and estimate the Spearman rank correlation coefficient. We find a correlation of $\rho_{s}\approx 0.4$ with P≪0.01. This correlation indicates that, during the flare, periods of spin-up tend to coincide with an increase in flux, while periods of spin-down tend to coincide with a decrease in flux and larger changes are more likely to occur together. We verify this result in several ways. First, we use Swift/BAT flux data from the same start dates (or the nearest future start dates, if the original had no data) instead of Fermi/GBM flux data to verify that the result is not caused by any systematics in Fermi/GBM data. Using Swift data, the correlation remains definitive (P≪0.01) with a slightly lower $\rho_{s}\approx 0.3$. Second, we gradually remove the points with the lowest change in flux, leaving only those observations that have the greatest change in flux. Statistically, this increases the probability of observing the moment of ingress/egress into the stream. When we do this, the correlation gradually increases up to $\rho_{s}\approx 0.5$ for the ∼100 points of greatest change in flux. When using Swift/BAT data, this causes the correlation coefficient to reach $\rho_{s}\approx 0.4\;\text{to}\;0.5$.

We also test whether frequency increases are more common at the beginning of flares, whereas frequency decreases are more common at the end of flares, irrespective of the scale of the change. Of 528 data points, the frequency decreases in 240 and increases in 288. In the same data, 373 points show a match between an increasing or decreasing frequency and the start or end of the flare, and 155 points show a mismatch. The odds ratio associated with the data is $6.0_{-1.1}^{+1.2}$. The probability evaluated using Fisher’s exact test of a table being this skewed toward matched increase/decrease arising from unrelated random variables due to random chance is ∼5 × 10^−22^, indicating a statistically significant connection between the change in flux and the change in frequency. The association can be an artifact of the secular evolution of the system, which has shown a frequency drift across the observation time (from a period of ∼685 to ∼669 s). To test this, we calculated the odds ratio for several arbitrary sets of start and end phases across the orbit. We select sets of two continuous, consecutive phase segments in the interval [0.2,0.8] of the same phase length as we used to calculate the odds ratio during the preperiastron flare. We find a weak skew toward an increase in frequency on earlier phases in all subsets (odds ratio to ∼1.2 to 1.5), but this result is statistically significant only in some subsets. We therefore reject the hypothesis that the observed correlation between the preperiastron flare and frequency changes can be explained by the secular evolution of the system.

We also estimate the robustness of this result using a Monte Carlo simulation. We randomly generate 10,000 new frequency-difference datasets based on the measured frequency difference and its associated uncertainty. In 90% of simulations, the resulting Spearman rank coefficient is greater than 0.35. In all of the simulations, the probability that a similarly skewed table would arise from a random selection of unrelated variables is 0.001 or less. We therefore conclude that the above results are robust. These results support our hypothesis that short-lived disk-like structures in GX 301–2 form during ingress and egress from the stream.
